# Protoplast Isolation and Shoot Regeneration from Protoplast-Derived Callus of *Petunia hybrida* Cv. Mirage Rose

**DOI:** 10.3390/biology9080228

**Published:** 2020-08-16

**Authors:** Hyun Hee Kang, Aung Htay Naing, Chang Kil Kim

**Affiliations:** Department of Horticultural Science, Kyungpook National University, Daegu 41566, Korea; a3220641@naver.com

**Keywords:** digestion time, osmoticum conditions, plating density, plant growth regulators, protoplast viability

## Abstract

Despite the increasing use of protoplasts in plant biotechnology research, shoot regeneration from protoplasts remains challenging. In this study, we investigated the factors involved in protoplast isolation, callus induction, and shoot regeneration in *Petunia hybrida* cv. Mirage Rose. The following conditions were found to be most optimal for protoplast yield and viability: 0.6 M mannitol, 2.0% cellulase, and 6 h digestion time. A plating density of 10 × 10^4^ protoplasts/mL under osmoticum condition (0.58 M mannitol) showed high microcolony viability in liquid culture. The Kao and Michayluk medium was found to be appropriate for callus proliferation from microcalli under a 16-h light photoperiod. Calli cultured in Murashige and Skoog medium containing 1.0 mg/L 6-benzylaminopurine and 0.2 mg/L 3-indole butyric acid showed the highest shoot regeneration frequency and number of shoots obtained per explant. Random amplification of polymorphic DNA analysis showed that the protoplast-derived shoots exhibited the same banding patterns as those of donor plants. Collectively, these findings can contribute to solving problems encountered in protoplast isolation and shoot regeneration in other petunia cultivars and related species. As the protocol developed by us is highly reproducible, it can be applied in biotechnology research on *P. hybrida* cv. Mirage Rose.

## 1. Introduction

Plant protoplasts are totipotent and can regenerate into various organs. In addition, they can easily take up foreign genetic material such as DNA, chromosomes, organelles, and viral particles [1,2]. Plant protoplasts have therefore garnered interest as experimental single cells in various fields of plant biotechnology, such as genetic manipulation, protoplast transient gene expression, plant gene functional characterization, and genome editing [3,4,5,6,7]. Despite the success of clustered regularly interspaced short palindromic repeats/CRISPR-associated 9 (CRISPR/Cas9)-mediated genome editing in several plant species using *Agrobacterium*-mediated transformation, off-target effects cause unwanted results. Indeed, target gene editing via ribonucleoprotein (RNP) complex delivery using protoplast-based technology is much less likely to produce off-target mutants compared with *Agrobacterium*-meditated transformation [8,9,10].

Various methods of isolating and culturing protoplasts in *Petunia hybrida* have been reported since a few decades ago [11,12,13,14,15,16]. However, shoot regeneration from a protoplast-derived callus has thus far remained challenging, although a few studies have reported genotype-dependent shoot regeneration from protoplast-derived calli [16], with most genotypes being minor or of no commercial importance. Indeed, successful protoplast isolation with high yield and reproducibility often requires an optimal digestion enzyme dose and digestion time [16,17,18]. In addition, the concentration of sucrose as a carbon source also plays a vital role in the success of protoplast culture [13,14,15,16]. Unfortunately, a protocol suitable for one cultivar might not be suitable for other cultivars. Therefore, optimization of the factors involved in protoplast isolation, callus induction, and shoot regeneration is necessary for each cultivar.

Research on genome editing, genetic manipulation, and *in vitro* propagation and conservation of endangered species requires genetically stable plants regenerated from a protoplast-derived callus. Unfortunately, genetic and chromosomal variations have been reported in protoplast-derived petunia plants [12,16,19]. Therefore, an optimized protocol is required to obtain genetically stable plants that can be beneficially used for genome editing via CRISPR/Cas9 RNP complexes.

In the present study, we optimized cellulase and mannitol concentrations and the digestion time for protoplast isolation as well as the plating density for microcalli formation. In addition, we investigated the effects of culture conditions (dark and light) and plant growth regulators (PGRs) on callus induction and shoot regeneration in *P. hybrida* cv. Mirage Rose. Moreover, random amplification of polymorphic DNA (RAPD) marker was used to assess the genetic stability of shoots regenerated from a protoplast-derived callus.

## 2. Materials and Methods

### 2.1. Plant Materials

*P. hybrida* cv. Mirage Rose seeds were germinated in hormone-free Murashige and Skoog (MS) medium [20] containing 3.0% sucrose and 0.8% agar. The cultures were placed under cool, white fluorescent light for 16 h (intensity, 35 µmol m^−2^ s ^−1^) at 25 °C ± 2 °C for 4 weeks. Finally, germinated shoots were subcultured in the same hormone-free MS medium for 4 weeks, and healthy leaves obtained from the 4-week-old plants were used for protoplast isolation.

### 2.2. Protoplast Isolation and Purification

Protoplast isolation and purification were performed as described by [21] with a few modifications. Briefly, leaves obtained from 4-week-old plants were gently chopped into approximately 0.3-mm segments. For plasmolysis, the segments were transferred to a 50-mL Falcon tube containing 10 mL of cell and protoplast washing (CPW) solution and different concentrations (0.4–0.7 M) of mannitol (Sigma-Aldrich, St. Louis, MO, USA) (Table S1) and incubated in a gyratory shaking incubator at 30 rpm for 2 h in the dark at 25 °C ± 2 °C. Next, the cell wall was digested by transferring the segments into an enzyme solution containing different concentrations (1.0%, 1.5%, 2.0%, and 2.5%) of Onozuka R-10 cellulase with 0.6% macerozyme R-10 (Yakult Pharmaceutical Ind. Co., Ltd., Tokyo, Japan), 0.01 M 2-(*N*-morpholino) ethanesulfonic acid (MES), and 0.2% bovine serum albumin (Sigma-Aldrich). The enzyme solutions were filter-sterilized through a 0.20 mm syringe filter (GVS, Sanford, ME, USA); the pH of all solutions was adjusted to 5.8 using NaOH. Explants were incubated in a gyratory shaking incubator at 30 rpm in the dark at 25 + 2 °C. Cell wall digestion was analyzed 6 h later. After enzymatic cell wall digestion, the solutions were filtered through a 125-µm nylon mesh into new Falcon tubes. The filtrate was added to 5 mL of autoclaved washing solution (Table S1) and centrifuged at 500 rpm for 5 min. Finally, the protoplasts were washed twice to remove the enzyme solutions.

For purification, approximately 1 mL of W5 washing solution was added to the protoplast pellet, which was then transferred to 10 mL of different sucrose concentrations (Table S1) using a Pasteur pipette. Centrifugation was performed at 1000 rpm for 10 min. Next, approximately 1 mL of floating viable protoplasts was transferred to 10 mL of 0.6 M sucrose, and centrifugation was again performed as described above. The protoplasts were collected in a 15-mL Falcon tube to test their viability and yield. In addition, after selecting optimal mannitol and cellulase concentrations, we evaluated the effects of different cell wall digestion times (2, 4, 6, and 8 h) on protoplast yield and viability.

### 2.3. Protoplast Yield and Viability Testing

Purified protoplasts were counted using a hemocytometer under an Olympus BX61 microscope (Olympus, Tokyo, Japan). The protoplast yield (*Y*) was expressed as the number of protoplasts per g of explant fresh weight (FW) as

*Y* = *n* × *p*/FW

where *Y* is the protoplast yield (protoplasts/g), *n* is the number of protoplasts/mL, *p* is the protoplast suspension volume (mL), and FW (g) is the leaf FW.

To evaluate protoplast viability, 0.5% fluorescein diacetate (FDA; Sigma-Aldrich) was dissolved in acetone. Then, 100 µL of protoplast suspension was stained with 1 µL of FDA and incubated in the dark for 5 min. The protoplasts were observed under a microscope. Viability was expressed as


Viability = (Number of green fluorescent protoplasts/Total number of observed protoplasts) × 100

The protoplast yield and viability were assessed on the basis of three independent isolations with three replicates per treatment.

### 2.4. Protoplast Culture

#### 2.4.1. Effect of Plating Density and Osmoticum Condition on Viability of Protoplast-Derived Microcolonies

Various densities of protoplasts (5, 10, and 15 × 10^4^ protoplasts/mL) were cultured in a liquid medium comprising Kao and Michayluk basal salt mixture, Gamborg B5 vitamin, 0.6 M mannitol, 1.0 mg L^−1^ of 6-benzylaminopurine (BA), 1.0 mg L^−1^ of 1-naphthaleneacetic acid (NAA), 0.1% MES, and 1.0% sucrose. The combination of BA 1.0 mg L^−1^ and NAA 1.0 mg L^−1^ was selected based on its optimal result in formation of microcolonies and microcalli in our preliminary experiments. After 2 weeks, floating protoplast-derived microcolonies were pipetted and transferred to fresh media under different osmoticum conditions (0.6, 0.58, and 0.56 M mannitol) for an additional 2 weeks by adjusting the medium volume to 4 mL. Finally, after 4 weeks of initial culture, the effect of the plating density and osmoticum condition on viability was evaluated. We used three independent plates with three replicates per treatment.

#### 2.4.2. Effect of Plating Density on Cell Division Frequency and Callus Formation

To determine the effect of plating density on cell division and callus formation, different densities (5 and 10 × 10^4^ protoplasts/mL) that showed higher viability were cultured in the same liquid medium as before. Every 2 weeks, floating protoplast-derived microcolonies were pipetted and transferred to fresh media under optimal osmoticum conditions (0.58 M mannitol) for an additional 2 weeks by adjusting the medium volume to 4 mL. Finally, after 4 weeks of culture, the cell division frequency observed at each plating density was recorded. We used three independent plates with three replicates per treatment.

According to the results of our preliminary experiment, the combination of BA 0.5 mg L^−1^ and NAA 0.5 mg L^−1^ was found to be optimal for callus formation from microcalli. Therefore, after a further 4 weeks of culture, microcalli induced from the cells were cultured in three Petri dishes (10 microcalli/Petri dish) containing a solid medium (MS media, Gamborg B5 vitamin, 0.5 mg L^−1^ of BA, 0.5 mg L^−1^ of NAA, and 3.0% sucrose) in the dark. After another 4 weeks of culture, the number of calli induced per Petri dish was recorded. We used three replicates per treatment.

#### 2.4.3. Effect of Different Media and Dark/Light Conditions on Callus Formation

Microcalli culture in solid MS medium resulted in a relatively low number of calli/Petri dish after 4 weeks of culture. Therefore, we further induced microcalli at a plating density of 10 × 10^4^ and cultured again in a different medium comprising MS, Kao and Michayluk full or half strength media. The three Petri dishes (10 microcalli/Petri dish) were placed in the dark. After 4 weeks of culture, we recorded the number of calli induced per Petri dish. In addition, we investigated the effects of culturing microcalli in Kao and Michayluk completely in the dark and in a 16-h/8-h light/dark cycle on callus induction. We used three replicates per treatment.

#### 2.4.4. Effect of Plant Growth Regulators on Shoot Induction from Callus

Calli were cultured in a shoot induction medium comprising MS, 3% sucrose, 0.8% plant agar, 0.5–2.0 mg L^−1^ 6-BA, and 0.1–0.5 mg L^−1^ 3-indole butyric acid (IBA) for 8 weeks. The cultures were placed under light for 16 h and subcultured every 4 weeks. Finally, the shoot regeneration frequency and the number of shoots/explant were calculated. Each treatment contained 10 calli with three replications.

### 2.5. Detection of Genetic Stability in Protoplast-Derived Shoots by RAPD

Total genomic DNA was isolated from the leaves of *in vitro* donor plants and protoplast-derived plants using the HiYield genomic DNA mini kit (Real Biotech Corporation, Taipei, Taiwan). RAPD was performed as described by [22]. To verify the banding pattern of the DNA samples, RAPD analysis was repeated at least three times for all the plants. The primers and polymerase chain reaction conditions used for RAPD are described in Supplemental file 2 (Table S2). The banding patterns were examined on 2% (*w/v*) agarose gel stained with ethidium bromide and photographed under ultraviolet light.

## 3. Statistical Analysis

Data were statistically analyzed using analysis of variance with SPSS version 11.09 (IBM Corporation, Armonk, NY, USA). Data were presented as means of three replications. Significance was determined using Duncan’s multiple range test, least significant difference test and *t*-test at *p* < 0.05.

## 4. Results

### 4.1. Effects of Mannitol and Cellulase Concentrations on Protoplast Isolation

**Figure 1:**
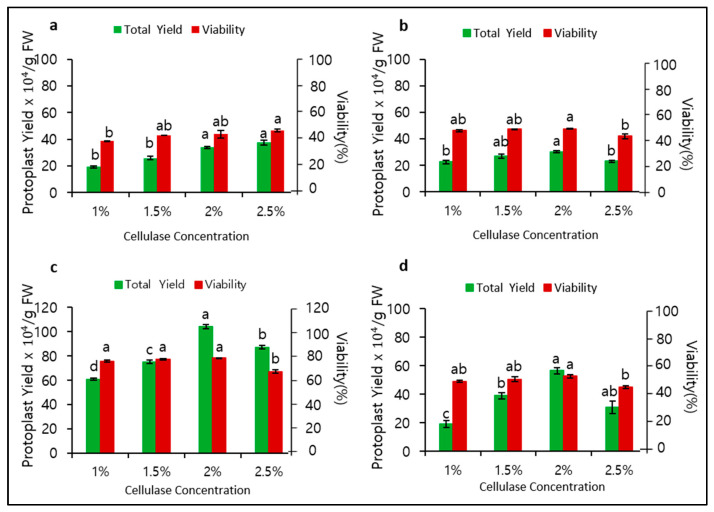
Effect of different mannitol and cellulase concentrations and 6 h digestion time on protoplast yield and viability: (**a**) 0.4, (**b**) 0.5, (**c**) 0.6, and (**d**) 0.7 M mannitol. Data represent the means of three replications. The bar indicates the standard deviation of three replications. Means with the same letters are not significantly different by Duncan’s multiple range test (*p* < 0.05).

To determine the role of mannitol and cellulase concentrations in protoplast isolation, we observed their significant effects on protoplast yield and viability. When 0.4 M mannitol was used for plasmolysis, the optimal cellulase concentration for cell wall digestion was found to be 2.5%, with a yield of 37.5 × 10^4^/gFW and viability of 45.6% (Figure 1a). At a mannitol concentration of 0.5 M, the optimal cellulase concentration was 2.0%, but the yield (30.3 × 10^4^/gFW) and viability (47.5%) were still relatively low (Figure 1b). Surprisingly, at a mannitol concentration of 0.6 M, both the yield and viability considerably increased at an optimal cellulase concentration of 2.0% to 104.1 × 10^4^/gFW and 73.3%, respectively (Figure 1c). Remarkably, at a mannitol concentration of 0.7 M, both yield and viability immediately decreased (Figure 1d). Therefore, 0.6 M mannitol and 2.0% cellulase were found to be most optimal for protoplast isolation in *P. hybrida* cv. Mirage Rose.

### 4.2. Effect of Digestion Time on Protoplast Isolation

**Figure 2:**
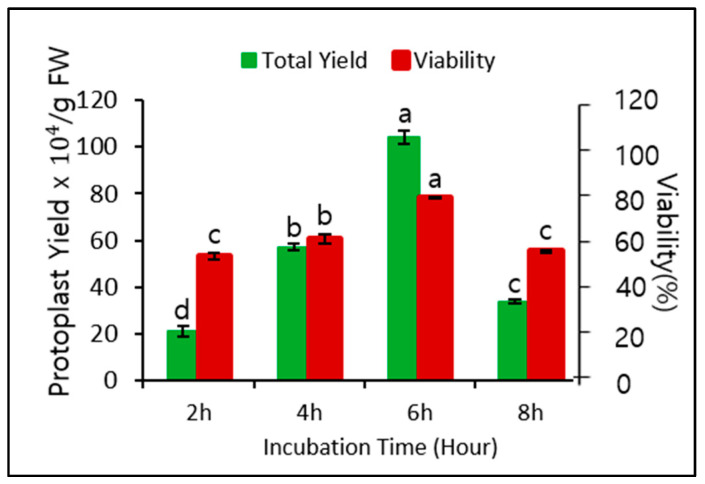
Effect of different digestion times (2, 4, 6, and 8 h) and 0.6 M mannitol and 2.0% cellulase on protoplast yield and viability. Data represent the means of three replications. The bar indicates the standard deviation of three replications. Means with the same letters are not significantly different by Duncan’s multiple range test (*p* < 0.05).

Application of 0.6 M mannitol for plasmolysis and 2.0% cellulase for cell wall digestion gave the best protoplast yield and viability when the digestion time was 6 h. Therefore, we assessed whether different digestion times affected the yield and viability. As shown in Figure 2, the digestion time significantly affected both yield and viability. The yield and viability were highest after 6 h of digestion, followed by 4 h of digestion, but both yield and viability after 2 and 8 h of digestion were relatively low. Therefore, a digestion time of 6 h was optimal for protoplast isolation.

### 4.3. Protoplast Culture

#### 4.3.1. Effect of Plating Density and Osmoticum Condition on Viability of Protoplast-Derived Microcolonies

**Figure 3:**
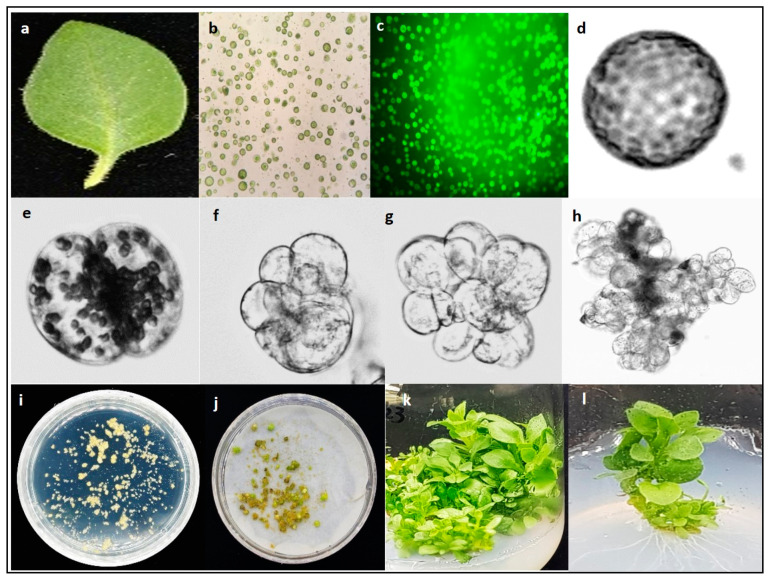
Protoplast isolation and shoot regeneration from a protoplast-derived callus of *Petunia hybrida* cv. Mirage Rose. (**a**) Fully expanded *in vitro* leaf used for protoplast isolation, (**b**) protoplasts isolated from leaf mesophyll cells, (**c**) protoplasts stained with fluorescein diacetate, (**d**) status of a single protoplast on day 1 in liquid culture, (**e**) first cell division on day 3, (**f**) second cell division on day 5, (**g**) third cell division on day 7, (**h**) microcolony formation, (**i**) microcalli formation from the microcolonies, (**j**) callus proliferation from microcalli, (**k**) shoot induction from the callus, and (**l**) *in vitro* rooted shoots derived from protoplasts.

Mesophyll protoplasts isolated from the leaves of *P. hybrida* cv. Mirage Rose (Figure 3a) were found to be spherical (Figure 3b), and compact, deep-green viable protoplasts were easily identified under a microscope (Figure 3c). However, when protoplasts were cultured in a liquid medium, the plating density and osmoticum condition significantly affected their viability. Application of 0.6 M mannitol in the liquid culture medium for 4 weeks resulted in low viability at all densities (5, 10, and 15 × 10^4^), especially at 15 × 10^4^, whereas decreasing the mannitol concentration from 0.6 M to 0.58 M after the first 2 weeks of initial culture significantly improved viability, with viability being the highest at a plating density of 10 × 10^4^ (Table 1). However, decreasing the mannitol concentration from 0.6 M to 0.56 M rapidly decreased the viability again. Overall, a plating density of 10 × 10^4^ in a liquid culture medium with 0.6 M mannitol for the first 2 weeks and 0.58 M mannitol for another 2 weeks results in the most optimal protoplast viability during culture.

#### 4.3.2. Effect of Plating Density on Callus Formation

**Figure 4:**
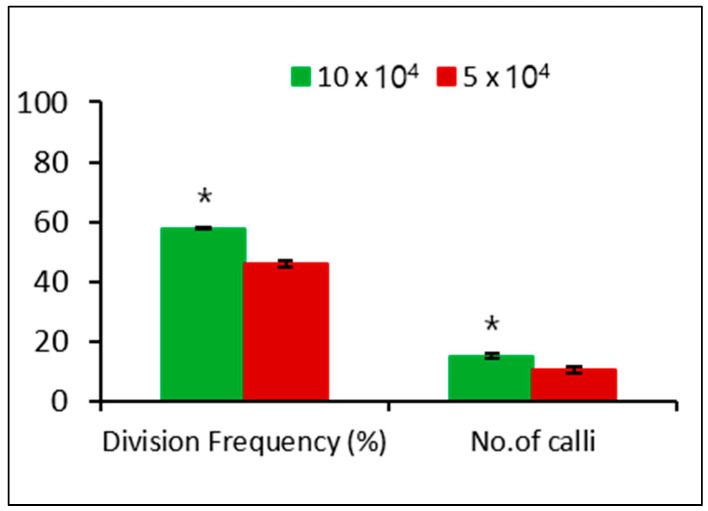
Effect of plating densities on cell division frequency and callus formation from microcalli in the dark. Data represent the means of three replications. The bar indicates the standard deviation of three replications. Mean with asterisk (*) is significantly different by the *t*-test (*p* < 0.05).

We investigated the effect of different plating densities on microcalli formation. After 3 days of culture, the single cell underwent division, followed by continuous mitotic cell division (Figure 3d–g), ultimately leading to microcolony formation after 2 weeks of culture (Figure 3h). The microcolony continued to form microcalli after 8 weeks of culture (Figure 3i), which converted to calli after culturing in solid MS medium for another 4 weeks in the dark (Figure 3j). The plating density had a significant effect on the cell division frequency and calli formation, with a low cell division frequency and callus formation observed at 5 × 10^4^ compared with that at 10 × 10^4^ (Figure 4).

**Figure 5:**
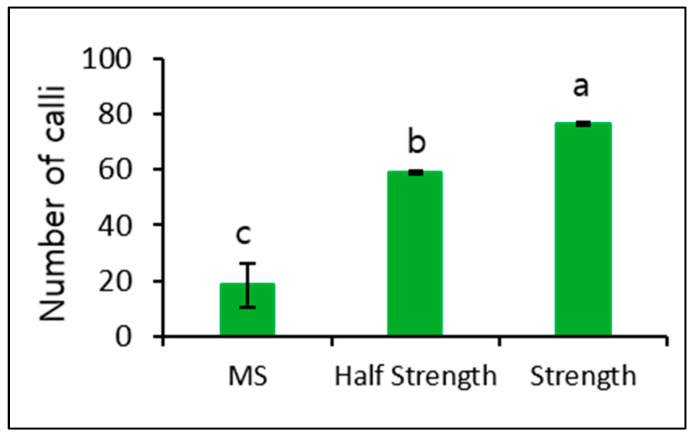
Effect of different culture media on callus formation from microcalli in the dark. Data represent the means of three replications. The bar indicates the standard deviation of three replications. Means with the same letters are not significantly different by the least significant difference test (*p* < 0.05).

The number of calli observed in MS medium was relatively low. Therefore, we further induced microcalli at a plating density of 10 × 10^4^ and cultured again using MS and both Kao and Michayluk media for 4 weeks in the dark. The number of calli induced in both Kao and Michayluk media was significantly higher than that induced in MS medium, with the highest number being observed in Kao and Michayluk full strength medium (Figure 5).

**Figure 6:**
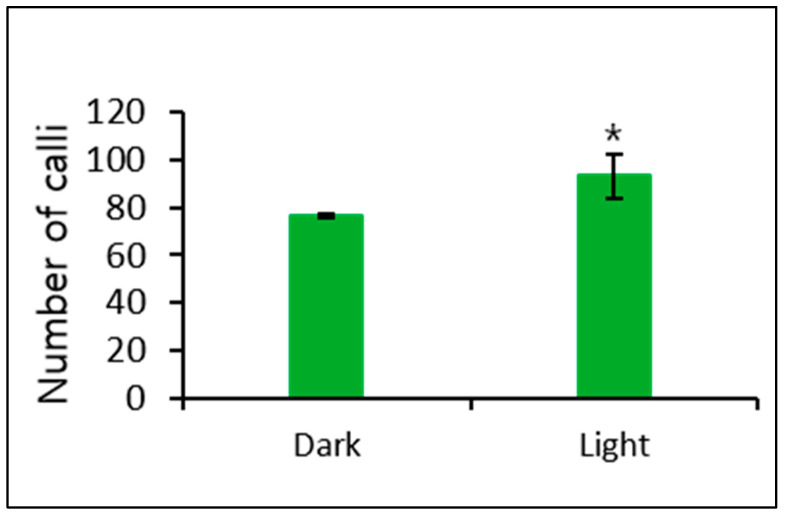
Effect of dark and light (16-h photoperiod) conditions on callus formation from microcalli cultured in Kao and Michayluk medium. Data represent the means of three replications. The bar indicates the standard deviation of three replications. Mean with asterisk (*) is significantly different by the *t*-test (*p* < 0.05).

In addition, we observed a significant effect of culturing in Kao and Michayluk medium completely in the dark and in a 16-h/8-h light/dark cycle on callus formation. More callus formation was induced when culturing in Kao and Michayluk medium in a 16-h/8-h light/dark cycle (Figure 6).

#### 4.3.3. Effect of Plant Growth Regulators on Shoot Regeneration from Protoplast-Derived Callus

The callus culture in MS basal medium did not regenerate any shoots and turned necrotic after 4 weeks of culture. A similar result was observed when a PGR combination of 0.5 mg/L 6-BA and 0.5 mg/L IBA was added to the medium. However, other treatments were able to induce shoot regeneration from the callus (Figure 3k). The combination of 1.0 mg/L 6-BA and 0.2 mg/L IBA showed higher shoot regeneration efficiency (53.3%), and higher number of shoots per explant (9.0) compared with those in other treatments (Table 2). Therefore, this combination was considered optimal for shoot regeneration from a protoplast-derived callus. In addition, the protoplast-derived shoots rooted and grew well in hormone-free MS basal medium (Figure 3l).

### 4.4. Analysis of Genetic Variation Using an RAPD Marker

**Figure 7:**
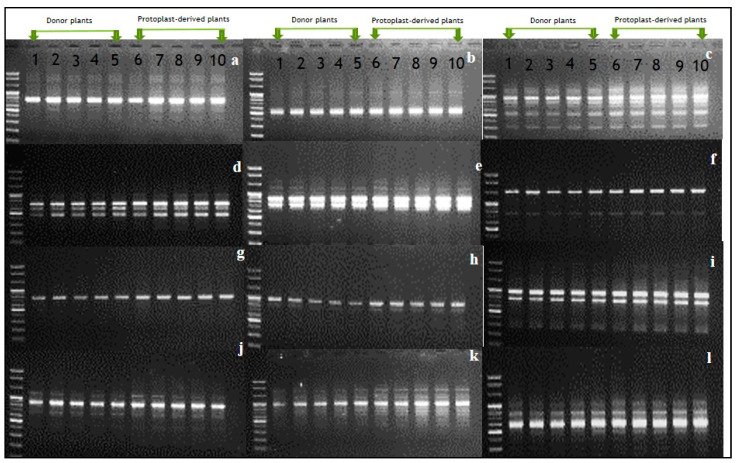
Detection of somaclonal variation between protoplast-derived plants and donor plants using random amplification of polymorphic DNA markers. (**a**) OPA-01, (**b**) OPA-02, (**c**) OPA-04, (**d**) OPA-09, (**e**) OPA-10, (**f**) OPA-12, (**g**) OPA-15, (**h**) OPB-2, (**i**) OPB-10, (**j**) OPB-11, (**k**) OPB-12, and (**l**) OPB-17.

RAPD results (Figure 7) indicated that of the 40 random primers used, 12 successfully produced scorable bands for both donor and protoplast-derived plants; however, the RAPD banding patterns differed depending on the primers used (Figure 7a–l). As the banding patterns observed in protoplast-derived shoots were identical to those in donor plants, it was likely that there may be no genetic variation among the plants tested.

## 5. Discussion

The use of protoplasts is currently increasing in CRISPR/Cas9-mediated genome editing studies because editing of a target gene via delivery of RNP complexes using protoplast-based technology has more potential to avoid off-target effects compared with *Agrobacterium*-mediated transformation [8,9,10]. However, protoplast-to-shoot regeneration remains challenging. Protoplast isolation and culture have been studied in *P. hybrida* [11,12,13,14,15,16], but few studies have reported shoot regeneration from a protoplast-derived callus owing to various factors restricted to cell division frequency, callus formation, and shoot regeneration. In addition, protoplast-derived petunia plants are often genetically varied [12,16,19]. In fact, researchers prefer genetically stable plants for studies on genome editing, genetic manipulation, and *in vitro* propagation and conservation of endangered species.

Therefore, in this study we optimized several factors that affect protoplast yield, viability, cell division, callus induction, and shoot regeneration in *P. hybrida* cv. Mirage Rose and validated genetic stability in the regenerated shoots using an RAPD marker. We also optimized the osmoticum condition and cell wall digestion, which usually affect protoplast yield and viability, using different concentrations of mannitol and cellulase. Optimal concentrations (0.6 M mannitol and 2.0% cellulase) led to high protoplast yield and viability, while a variation of these concentrations significantly affected protoplast yield and viability. The protoplast yield and viability were low at low (0.4 an 0.5 M) and high mannitol concentrations (0.7 M) because (1) too many water molecules entered through the plasma membrane, bursting the protoplasts in a hypotonic solution, or (2) too many water molecules were left in the cell, shrinking the protoplasts in a hypertonic solution via osmosis. A mannitol concentration of 0.6 M provided an appropriate osmoticum environment for adequate plasmolysis. Protoplast isolation with the same mannitol concentration (0.6 M) has been previously reported in other petunia cultivars [15,16] reported the highest yield at 2.0% cellulase in different petunia cultivars, which is similar to our results, and 1.0% cellulase has been used to obtain high yields in other petunia cultivars [13,15] despite a relatively low yield in *P. hybrida* cv. Mirage Rose. Although the mechanism of cell wall degradation by cellulase is complex, it seemed that a low cellulase concentration (1.0%) is not sufficient to digest cellulose in the plant cell wall, therefore releasing fewer protoplasts. The low protoplast yield after digestion for 2 or 4 h compared with 6 h is due to the shortage of time to break down the cell wall, especially at 2 h. In addition, the significant decrease in the protoplast yield after digestion for 8 h is probably due to the damage to the plasma membrane. In fact, optimal digestion times often vary depending on cellulase concentration and plant species used [11,13,16,18,23,24,25,26,27]. Therefore, it is necessary to optimize the digestion time to obtain a high protoplast yield.

Protoplast-derived microcolonies should be viable throughout the protoplast culture in order to undergo microcalli formation. The plating density and osmoticum condition affect the viability of protoplast-derived microcolonies. In this study, attaining of lower microcolony viability at a higher plating density (15×10^4^) compared with those at low plating densities (5 × 10^4^ and 10 × 10^4^) could be due to high phenol accumulation in the culture media. A similar finding was reported in chrysanthemum [20]. In petunia cultivars, plating densities such as 15 × 10^4^ [16], 50 × 10^4^ [13], and 5 × 10^4^ [15] have been used to achieve cell division and microcalli formation; however, these authors used only one plating density in their studies, and they did not examine the significant effect of the plating density on microcolony viability.

In addition, after 2 weeks of culture, adjusting the osmoticum condition by slightly decreasing the mannitol concentration (from 0.6 M to 0.58 M) also improved microcolony viability, but the mannitol concentration should be optimized because too low mannitol concentration (0.56 M) rapidly decreases microcolony viability, regardless of planting density. It is likely that the optimal osmoticum condition is changing depending on cell division progress. Attaining of the higher cell division frequency and callus induction at 10 × 10^4^ compared with that at 5 × 10^4^ could be due to the slightly higher microcolony viability in the former than in the latter.

MS full- and half-strength media have been successfully used to induce callus formation from protoplast-derived microcalli in several plant species, including petunia species [13,16,21,28]. However, previous studies did not investigate the role of different culture media in callus formation. In this study, we found Kao and Michayluk to be significantly higher than MS and Kao and Michayluk half strength medium, thus, it can be suggested that Kao and Michayluk medium is most suitable for callus proliferation from protoplast-derived microcalli in petunia species. In addition, a 16-h/8-h light/dark cycle is better than a dark condition for callus formation.

The callus was unable to regenerate shoots on hormone-free MS medium, but they can regenerate in culture media containing different concentrations of 6-BA and IBA, indicating that PGRs are required to induce shoots from a protoplast-derived callus. However, no shoots regenerated in culture media containing 0.5 mg/L 6-BA and 0.5 mg/L IBA, probably because of the cytokinin and auxin balance, which is generally used for callus induction and proliferation. Of all treatment combinations, 1.0 mg/L BA and 0.2 mg/L IBA is the most suitable for shoot regeneration from a protoplast-derived callus. Genetic stability of *in vitro* regenerated plants has been detected in several plant species using RAPD marker [22,28,29,30]. In this study, as RAPD analysis showed occurrence of the same banding patterns in protoplast-derived shoots compared with those in donor plants. Perhaps it could be due to absence of genetic variation in the regenerated plants, while similar studies on petunia cultivars reported genetic variation [12,16,19]. Rahmani et al. [28] also reported that protoplast-derived shoots of *Albizia julibrissin* are genetically stable. Therefore, our protocol would be valuable for application in various biotechnology studies on *P. hybrida* cv. Mirage Rose.

## 6. Conclusions

We successfully regenerated shoots from protoplasts by optimizing several factors affecting the long process of protoplast isolation to shoot regeneration. The optimal conditions for the best protoplast yield and viability are 0.6 M mannitol, 2.0% cellulase, and 6 h of digestion. In addition, a plating density of 10 × 10^4^ with 0.58 M mannitol (osmoticum condition) favors high viability of microcolonies during liquid culture, and microcalli culture in the Kao and Michayluk medium under light for 16 h significantly proliferate calli. The highest shoot regeneration frequency and number of shoots per explant are observed in the culture medium containing 1.0 mg/L BA and 0.2 mg/L IBA. The regenerated shoots are genetically stable in comparison with donor plants. Our findings will aid researchers working on protoplast isolation and shoot regeneration in other petunia cultivars and related species. We highly recommended that our protocol is very reproducible and can be applied in biotechnology research on *P. hybrida* cv. Mirage Rose.

## Figures and Tables

**Table 1:** Effect of plating density and osmoticum condition on microcolony viability after 28 days in liquid culture.

	Microcolony Viability		
Plating Density (×10^4^)	Mannitol (0.6 M)	Mannitol (0.58 M)	Mannitol (0.56 M)
	Day 28	Day 28	Day 28
15	20.1 ± 1.7 f	53.2 ± 0.2 b	35.7 ± 1.4 c
10	41.1 ± 0.6 cde	61.5 ± 1.0 a	36.5 ± 1.6 de
5	46.2 ± 2.6 c	57.7 ± 2.7 ab	42.2 ± 1.2 cd

Data represent the means ± standard deviation of three replications. Means with the same letters are not significantly different by Duncan’s multiple range test at *p* < 0.05.

**Table 2:** Effect of plant growth regulators on shoot regeneration from a protoplast-derived callus of *Petunia hybrida* cv. Mirage Rose.

PGR (mg L^−1^)		Regeneration (%)	No. of Shoots/Explant
6-BA	IBA		
0	0	0 d	0 e
0.5	0.5	0 d	0 e
1	0.1	40 b	1.8 c
1	0.2	53.3 a	9.0 a
1	0.5	20 c	1.0 d
1.5	0.5	40 b	3.5 b
2.0	0.5	44.4 b	4.0 b

Data represent the means of three replications. Means with the same letters are not significantly different by Duncan’s multiple range test at *p* < 0.05.

## Supplementary Materials

The following are available online at https://www.mdpi.com/2079-7737/9/8/228/s1. Table S1: Chemical solutions used for plasmolysis and purification of protoplasts, Table S2: Primers, sequences, and PCR conditions used for RAPD analysis.
