# Supplementary material for: Protoplast Isolation and Shoot Regeneration from Protoplast-Derived Callus of Petunia hybrida Cv. Mirage Rose
Source: Biology (Basel). 2020 Aug 16;9(8):228. doi: 10.3390/biology9080228 (PMC7465674; doi:10.3390/biology9080228)
Supplement: Supplementary file 1 [file biology-09-00228-s001.pdf]

Table S1. Chemical solutions used for plasmolysis and purification of protoplasts.

**For plasmolysis CPW solution (mg/L)**	
KI	0.16
$CuSO_4 \cdot H_2O$	0.025
KH_2PO_4	27.2
KNO_3	101
$CaCl_2 \cdot 2H_2O$	1480
$MgSO_4 \cdot 7H_2O$	246
Plasmolysis with different mannitol concentrations	
CPW+0.4M Mannitol	
CPW+0.5M Mannitol	
CPW+0.6M Mannitol	
CPW+0.7M Mannitol	
**For purification** W5 solution (g/L)	
5mM glucose	0.991
154mM NaCl	8.99
125mM $CaCl_2$	18.37
5mM KCl	0.373
0.1% MES	1
Different mannitol concentrations used for purification	
W5+0.3M Mannitol	
W5+0.4M Mannitol	
W5+0.5M Mannitol	
W5+0.6M Mannitol	
Different sucrose concentrations used for isolation of viable protoplasts	
CPW+0.4M Sucrose	
CPW+0.5M Sucrose	
CPW+0.6M Sucrose	
CPW+0.7M Sucrose	

Table S2. Primers, sequences, and PCR conditions used for RAPD analysis.

**Primers**	**Sequence**	**Temperature (°C)**
OPA-01	CAG GCC CTT C	33
OPA-02	TGC CGA GCT G	33
OPA-03	AGT CAG CCA C	28.9
OPA-04	AAT CGG GCT G	28.9
OPA-05	AGG GGT CTT G	28.9
OPA-06	GGT CCC TGA C	33
OPA-07	GAA ACG GGT G	28.9
OPA-08	GTG ACG TAG G	28.9
OPA-09	GGG TAA CGC C	33
OPA-10	GTG ATC GCA G	28.9
OPA-11	CAA TCG CCG T	28.9
OPA-12	TCG GCG ATA G	28.9
OPA-13	CAG CAC CCA C	33
OPA-14	TCT GTG CTG G	28.9
OPA-15	TTC CGA ACC C	28.9
OPA-16	AGC CAG CGA A	28.9
OPA-17	GAC CGC TTG T	28.9
OPA-18	AGG TGA CCG T	28.9
OPA-19	CAA ACG TCG G	28.9
OPA-20	GTT GCA ATC C	28.9
OPB-01	GTT TCG CTC C	28.9
OPB-02	TGA TCC CTG G	28.9
OPB-03	CAT CCC CCT G	33

OPB-04	GGA CTG GAG T	28.9
OPB-05	TGC GCC CTT C	33
OPB-06	TGC TCT GCC C	33
OPB-07	GGT GAC GCA G	33
OPB-08	GTC CAC ACG G	33
OPB-09	TGG GGG ACT C	33
OPB-10	CTG CTG GGA C	33
OPB-11	GTA GAC CCG T	28.9
OPB-12	CCT TGA CGC A	28.9
OPB-13	TTC CCC CGC T	33
OPB-14	TCC GCT CTG G	33
OPB-15	GGA GGG TGT T	28.9
OPB-16	TTT GCC CGG A	28.9
OPB-17	AGG GAA CGA G	28.9
OPB-18	CCA CAG CAG T	28.9
OPB-19	ACC CCC GAA G	33
OPB-20	GGA CCC TTA C	28.9

PCR condition: 95℃ for 2min, followed by 35cycles of 95 ℃ 20s, Temperature (℃) 53s, 72 ℃ for 1min and 72 ℃ 5min.
